# Improved serodiagnosis of *Trypanosoma vivax* infections in cattle reveals high infection rates in the livestock regions of Argentina

**DOI:** 10.1371/journal.pntd.0012020

**Published:** 2024-06-26

**Authors:** Iván Alejandro Bontempi, Diego Gustavo Arias, Graciela Verónica Castro, Luz María Peverengo, Genaro Francisco Díaz, Martín Allassia, Gonzalo Greif, Iván Marcipar

**Affiliations:** 1 Laboratorio de Tecnología Inmunológica, Facultad de Bioquímica y Ciencias Biológicas, Universidad Nacional del Litoral, Santa Fe, Argentina; 2 Facultad de Ciencias Médicas, Universidad Nacional del Litoral, Santa Fe, Argentina; 3 Laboratorio de Enzimología Molecular, Instituto de Agrobiotecnología del Litoral (CONICET-UNL), Santa Fe, Argentina; 4 Cátedra de Bioquímica Básica de Macromoléculas, Facultad de Bioquímica y Ciencias Biológicas, Universidad Nacional del Litoral, Santa Fe, Argentina; 5 Facultad de Ciencias Veterinarias, Universidad Nacional del Litoral, Santa Fe, Argentina; 6 Laboratorio de Interacciones Huésped Patógeno-UBM, Institut Pasteur de Montevideo, Montevideo, Uruguay; Institut de recherche pour le developpement, FRANCE

## Abstract

Bovine trypanosomosis, caused by *Trypanosoma vivax*, currently affects cattle and has a significant economic impact in sub-Saharan Africa and South America. The development of new diagnostic antigens is essential to improve and refine existing methods. Our study evaluated the efficacy of two recombinant antigens in detecting specific antibodies in cattle. These antigens are derivatives of an invariant surface glycoprotein (ISG) from *T*. *vivax*. A fraction of a previously described antigen (TvY486_0045500), designated TvISGAf, from an African strain was evaluated, and a new ISG antigen from an American isolate, TvISGAm, was identified. The two antigens were expressed as fusion proteins in *Escherichia coli*: TvISGAf was fused to the MBP-His-tag, and TvISGAm was obtained as a His-tag fused protein. An ELISA evaluation was conducted using these antigens on 149 positive and 63 negative bovine samples. The diagnostic performance was enhanced by the use of a combination of both antigens (referred to as TvISG-based ELISA), achieving a sensitivity of 89.6% and specificity of 93.8%. Following the validation of the TvISG-based ELISA, the seroprevalence of *T*. *vivax* infection in 892 field samples from cattle in the central region of Argentina was determined. The mean seroprevalence of *T*. *vivax* was 53%, with variation ranging from 21% to 69% among the six departments studied. These results support the use of the TvISG ELISA as a valuable serological tool for the detection and monitoring of *T*. *vivax* infection in cattle. Furthermore, we report for the first time the seroprevalence of *T*. *vivax* in Argentina, which highlights the widespread endemic nature of the disease in the region. In order to effectively manage the increasing spread of *T*. *vivax* in the vast livestock production areas of South America, it is essential to implement consistent surveillance programs and to adopt preventive strategies.

## Author summary

Bovine trypanosomosis is a disease that threatens cattle health and agriculture in South America and Africa. It is mainly caused by the parasite *Trypanosoma vivax*. Diagnosis of this infection has been challenging, hampering efforts to monitor and control outbreaks. In this study, we developed a more accurate and sensitive test for detecting *T*. *vivax* in cattle. Using recombinant proteins tailored to parasite strains in the Americas, this new test can identify infection rates and patterns within cattle herds and regions. By analyzing over 800 cattle blood samples from Argentina ranchlands, we discovered over 50% seropositivity, indicating a far higher prevalence than previously recognized. These results highlight the urgent need for surveillance and preventive strategies, although diagnostic tools provide a means for tracking regional spread. By enhancing the diagnosis of this livestock disease, this study lays the foundation for protecting cattle health and associated livelihoods across at-risk communities in the developing world. This study exemplifies how molecular innovations can address veterinary challenges and promote food security within resource-constrained farming systems.

## Introduction

African Animal Trypanosomosis (AAT) represents a significant threat to the health and productivity of livestock in Africa and Latin America [1]. In South America, *Trypanosoma vivax (T*. *vivax)* is the primary causative agent of AAT and is mainly transmitted mechanically by hematophagous flies of the genera Stomoxys and Tabanus in the absence of the biological vector, the tsetse fly [2]. In this region, AAT poses a potential risk to nearly 350 million cattle, with studies on outbreaks in the Pantanal region of Brazil and Bolivia estimating a potential loss of US$160 million [3].

Among the tsetse fly-transmitted trypanosomes (*Salivaria*), *T*. *vivax* is phylogenetically positioned in the early branch [4,5] and belongs to the subgenus *Duttonella*, which causes severe wasting disease primarily in cattle, but can also infect sheep, goats, camels, horses, and buffalo [1,6]. Acute *T*. *vivax* infection is characterized by anemia, fever, poor body condition, and abortion. Occasionally, the disease progresses to more severe disease, such as severe neurological symptoms, and can be fatal [7–9]. Chronic *T*. *vivax* infection leads to progressive anemia, weight loss, and reproductive failure, resulting in a marked reduction in milk production [10,11]. However, infected cattle can be asymptomatic [12], and if the infection is left untreated, the animals can become asymptomatic carriers, potentially spreading and perpetuating the disease throughout the herd [13]. Although the earliest reports in South America date back to French Guiana in 1919 [14], the first outbreak in Argentina was recorded in 2006 in Formosa, northeastern Argentina [15]. Argentina is one of the world’s leading beef exporters, and the northeastern and central-eastern regions are important cattle production areas. Despite initial reports in Argentina, *T*. *vivax* was only recently identified by molecular methods in 2018 [16] and was associated with acute disease in dairy cattle in the Pampas region, with significant economic impact [17].

Diagnosis of *T*. *vivax* trypanosomosis must rely on highly sensitive and specific methods capable of detecting cryptically infected animals that may serve as healthy carriers, while also distinguishing between *T*. *vivax* and other trypanosomes, such as *T*. *theileri* and *T*. *evansi*. Molecular assays, such as conventional PCR, have successfully detected *T*. *vivax* [4,11,17]. However, these assays are expensive and require specialized laboratories to perform, making them inaccessible to field veterinarians and dairy farm owners. The use of serologic techniques has emerged to assess the presence of *Trypanosoma* in large sample volumes at an affordable cost. Serologic diagnostic techniques for AAT are based on the detection of antibodies to parasites using an indirect fluorescent antibody test (IFAT), as described by Luckins and Mehlitz in 1978 [18]. Although the IFAT is sensitive and specific, it is not quantitative, requires fluorescence-enabled microscopes, and lacks standardized antigen preparation. Whole trypanosomal lysate has also been used for the diagnose AAT by ELISA [19]. Although it has high sensitivity and specificity, it also has the disadvantage of requiring constant parasite production, making its preparation and standardization challenging. Assays using recombinant proteins have eliminated the need for live parasite preparation. Several antigens have been proposed for the diagnosis of bovine trypanosomosis, including MyxoTLm [20], GM6 [21,22], variant surface glycoproteins (VSGs) [23] and invariant surface glycoproteins (ISGs) [24,25].

ISGs are conserved antigens of unknown function that are expressed at relatively low levels on the trypanosome membrane and have no known antigenic variation [26]. In recent years, ISGs have gained attention for their conserved functionality in diagnostic roles, detecting *T*. *brucei gambiense* in human African trypanosomiasis [27], and for both *T*. *congolense* and *T*. *vivax* in AAT [24,25]. Fleming et al. (2016) identified an ISG with high diagnostic potential, designated TvY486_0045500 [24]. However, it was amplified from an African strain and evaluated using sera from African cattle infected with *T*. *vivax*. In the present study, we developed a diagnostic method using the TvY486_0045500 antigen and ISG from an American *T*. *vivax* isolate. We evaluated its performance using sera from cattle infected with the American *T*. *vivax* strains. In addition, we used the developed assay to determine the prevalence of *T*. *vivax* in the dairy basin of Argentina.

## Materials and methods

### Ethics statement

The study was conducted in strict accordance with the recommendations of the Argentinean National Council of Animal Experimentation and approved by the Research Ethics and Safety Committee of the Facultad de Bioquimica y Ciencias Biologicas of the Universidad Nacional del Litoral (process number: CE2021-02). Handling and sampling of cattle were performed by trained personnel, with animal safety and welfare as priority and in strict accordance with good animal practice guidelines. by the Animal. The authors confirm that the ethical policies of the journal, as noted on the journal’s author guidelines page, have been adhered to.

### Materials

Bacteriological medium was purchased from Britania Laboratories. *Taq* and *Pfu* DNA polymerase, T4 DNA ligase, and restriction enzymes were purchased from Promega and Thermo Fisher Scientific. Amylose resin was purchased from New England Biolabs. Ni^2+^-HiTrap chelating HP column was purchased from GE Healthcare. All other reagents and chemicals were of the highest quality and were commercially available from Sigma-Aldrich and Merck.

### Bacteria, plasmids, genetic material and *T*. *vivax* cells

*Escherichia coli* TOP10 F´ (Invitrogen) and *E*. *coli* BL21 (DE3) (Novagen) cells were used for routine plasmid construction and expression experiments, respectively. The pGEM-T Easy vector (Promega) was used for cloning and sequencing. The expression vectors used were pET28 (for N- and C-terminal His-tag protein generation, Novagen) and pMOMAL (a homemade-derived plasmid from pMAL C2 with recombinant His-tags and MBP-tags). Genomic DNA from *T*. *vivax* was obtained using the Wizard Genomic DNA Purification Kit (Promega). DNA manipulation, *E*. *coli* culture, and transformation were performed according to the standard protocols [28].

### Molecular cloning

Based on the available information on *T*. *vivax* genome sequences (https://tritrypdb.org/), ISG-encoding sequences were amplified by PCR using genomic DNA from the African strain of *T*. *vivax* Y486 (TvY486_0045500 - https://tritrypdb.org/tritrypdb/app) and *T*. *vivax* isolated in Argentina (tig00000163 –[29]). PCR was performed using genomic DNA and specific primer pairs (S1 Table) under the following conditions: 95°C for 10 min; 30 cycles of 95°C for 30 s, 55–65°C for 30 s, 72°C for 1.5 min, and 72°C for 10 min. The PCR product was them purified and ligated into the pGEM-T Easy vector, and its fidelity and identity were confirmed by complete DNA sequencing (Macrogen, South Korea). The resulting pGEM-T Easy constructs were digested with the appropriate restriction enzymes and the purified amplicons were ligated into different expression vectors using T4 DNA ligase (Promega) for 16 h at 4°C. Truncated versions of the African and American ISG sequences, TvISGAf and TvISGAm, were cloned into the pMOMAL and pET28 plasmids, respectively. Competent *E*. *coli* BL21 (DE3) cells transformed with the construct were selected in agar plates containing Lysogeny Broth (LB; 10 g L^-1^ NaCl, 5 g L^-1^ yeast extract, 10 g L^-1^ peptone, pH 7.4) supplemented with ampicillin (100 μg mL^-1^) or kanamycin (50 μg mL^-1^), as appropriate. Plasmid DNA was prepared and subsequent restriction treatment was performed to verify the correctness of the construct.

### Overexpression and purification of the recombinant protein

A single colony of *E*. *coli* BL21 (DE3), transformed with each recombinant plasmid, was selected. Overnight cultures were diluted 1/100 in fresh LB medium supplemented with the appropriate antibiotic and grown under identical conditions to the exponential phase, with an OD_600_ of 0.6. Expression of the respective recombinant proteins was performed with 0.25 mM IPTG for 16 hours at 23°C. Cells were harvested and stored at -20°C until further use.

Purification of each recombinant protein was performed by IMAC using a 1 mL Ni^2+^-HiTrap chelating HP column (GE Healthcare). Briefly, the bacterial pellet was resuspended in binding buffer (20 mM Tris–HCl pH 7.5, 400 mM NaCl, and 10 mM imidazole) and disrupted by sonication using a high-intensity ultrasonic processor (Vibra-cell TM VCX-600; Sonics & Materials Inc.). The lysate was centrifuged (10 000 ×*g* for 30 min) to remove cell debris. The resulting crude extract was applied to a column equilibrated with binding buffer. After washing with 10 bead volumes of the same buffer, the recombinant protein was eluted with elution buffer (20 mM Tris–HCl pH 7.5, 400 mM NaCl, 300 mM imidazole). In addition, TvISGAf isolated from IMAC chromatography (as an MBP fusion protein) was purified by amylose affinity chromatography (using a 5 mL amylose column, New England Biolabs), as a complementary step, according to the manufacturer’s instructions. To assess the potential for cross-reactivity with the fusion protein MBP, MBP was expressed and purified using the pMOMAL plasmid. Fractions containing pure proteins were pooled, concentrated, dialyzed (using 20 mM Tris-HCl buffer pH 8.0, 200 mM NaCl, and 1 mM EDTA), and frozen at -80°C in 20% (v/v) glycerol.

### Protein methods and immunodetection

Protein concentration was determined by the method described by Bradford [30] using bovine serum albumin (BSA) as a standard.

Immunodetection experiments were adapted from a previously described method for *T*. *cruzi* [31]. Briefly, polyclonal antibodies against TvISGAf and TvISGAm were obtained by immunizing mice with 10 μg of purified recombinant protein at two-week intervals, as previously described [32]. *T*. *vivax* cells of the Y486 strain, obtained from mice infected for four days, were pelleted and washed twice for 15 minutes at room temperature with sterile PBS buffer and then fixed in 4% (v/v) formaldehyde. After washing, they were permeabilized and blocked for 30 min in a medium containing PBS with the addition of 0.1% (v/v) Triton X-100 and 3% (w/v) BSA. The primary antibodies used were either mouse anti-TvISAf sera (1/100) or mouse anti-TvISGAm sera (1/100), along with rabbit anti-TccPx polyclonal antibodies (1/100 dilution). Slide wells were washed three times with blocking solution (3x50 μl). Secondary antibodies diluted 1/100 in blocking buffer (20 μl) were added to the slide wells and incubated for 60 min. FITC-conjugated goat anti-rabbit or Alexa 680-conjugated goat anti-mouse antibodies were used as secondary antibodies. DAPI (20 μl/slide well), diluted in PBS (final concentration 0.5 μg/ml), was added to each slide well. Slides were examined with a confocal microscope (Leica).

### Parasitological diagnosis

Blood was collected from the jugular vein of dairy cattle. For the diagnosis of *Trypanosoma* spp., blood samples were immediately transferred to glass microhematocrit capillary tubes containing sodium heparin (80 U/mL, Biocap) within six hours of collection. The capillary tubes were then centrifuged in a microhematocrit centrifuge at 9 000 rpm for 5 min. Packed cell volumes were them determined. Trypanosome motility was assessed according to previously established protocols [33]. A molecular diagnosis was conducted to confirm the diagnosis of *T*. *vivax* infection. For the purpose of molecular diagnosis, blood samples were collected using guanidine/EDTA and stored at 4°C until further analysis. Genomic DNA was extracted from 200 μL of blood using the High Pure PCR Template Preparation Kit (Roche), following the manufacturer’s instructions. PCR amplification targeting sequences corresponding to the catalytic domain of CatL-like (cdCatL-like) enzymes was performed using the oligonucleotide primers TviCatL1 and DTO155 [4]. PCR assays were performed in 25 μL reaction volumes containing 1X reaction buffer (100 mM Tris-HCl, 50 mM KCl, pH 8.8), 0.2 mM dNTP, 1–4 mM MgCl_2_, 0.4 μM of each primer, 0.625 U Taq polymerase (Promega), and 5 μL of DNA samples. The PCR products were visualized using 2% agarose gel electrophoresis, stained with GelRed (Millipore), and observed under UV light.

In addition, a 383-bp fragment of the mammalian Cyt b gene was amplified for all samples that were negative by the molecular method [34]. This was done to assess the DNA integrity and quality, including the presence of inhibitors. Samples that did not amplify were excluded from subsequent data analysis.

### Positive and negative serum samples

The serum panel consisted of 212 serum samples, 149 positive and 63 negative, from dairy cattle, mainly the Holstein and Jersey breeds. Positive serum samples were collected from naturally infected cattle on either day 15 or 21 after confirmation by microscopic examination of thin blood smears, buffy coat, and TviCatL-PCR [4]. Negative serum samples were obtained from cattle in non-endemic areas where no cases of *T*. *vivax* have been reported. For the preliminary antigen evaluation, and cross-reactivity with the fusion protein MBP, we randomly selected 30 positive and 30 negative samples from the entire panel. Finally, serum samples from cattle infected with *Babesia bovis* (n = 20*)*, *Anaplasma marginale* (n = 20), and *T*. *theileri* (n = 8) were used as negative controls for infections caused by unrelated hemoprotozoan parasites. These infections were confirmed by observation of parasitemia and detection of specific antibodies in cattle blood and sera.

### Field survey

**Fig 1:**
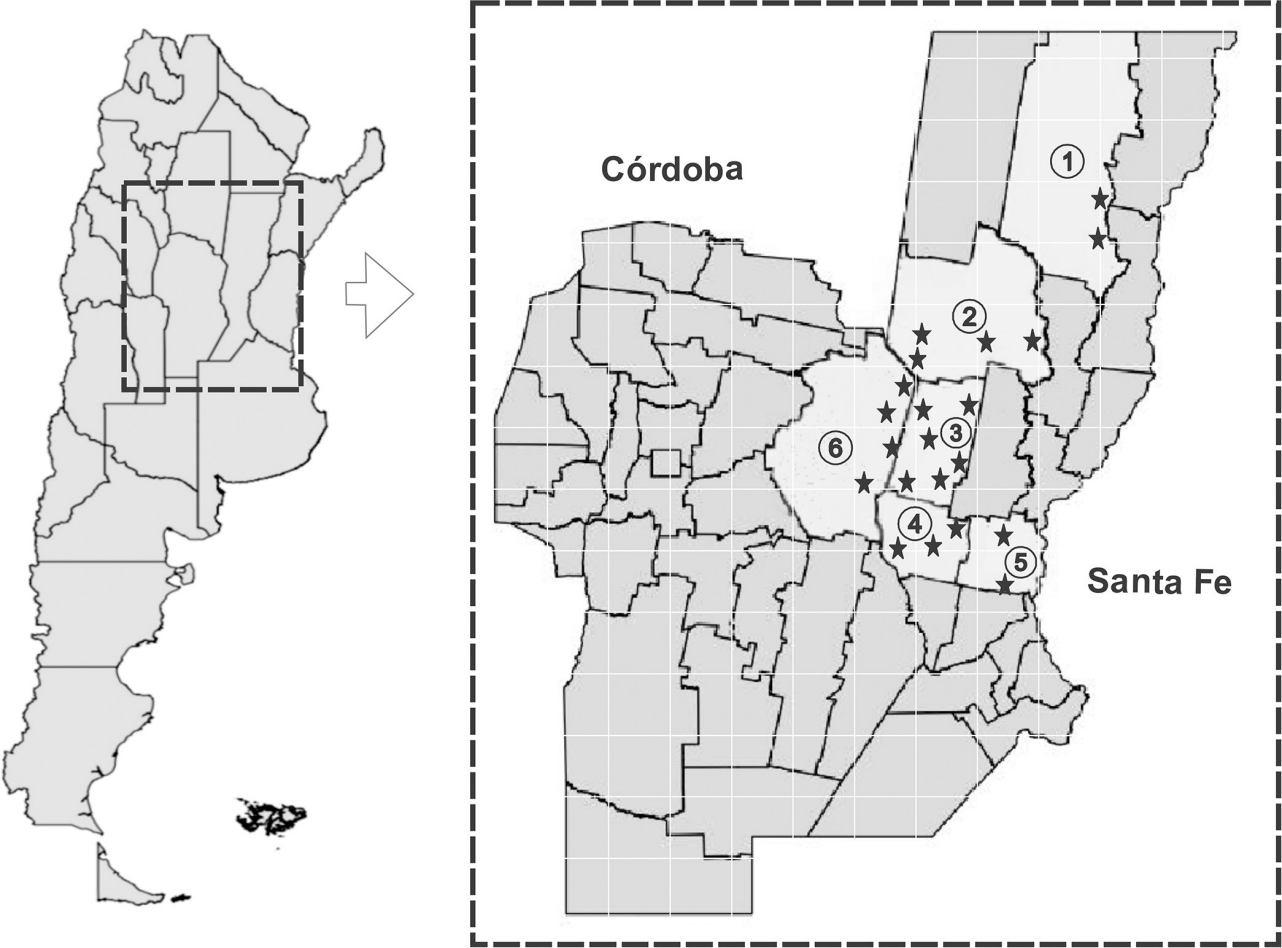
Map of Argentina showing the location of the two provinces included in the 2021–2023 study. **The dentification number (IN)** indicates the departments: ①: Vera, ②: San Cristóbal, ③: Castellanos, ④: San Martin, ⑤: San Jeronimo, ⑥: San Justo. **Stars** indicate cities, towns, villages, communes. Maps were generated using the free open-source Geographic Information System software. Maps were generated using the Python 3.10 programming language (https://www.python.org/downloads/release/python-3100/); shape files are freely available at https://www.ign.gob.ar/NuestrasActividades/InformacionGeoespacial/CapasSIG.

An extensive field survey was conducted in the provinces of Santa Fe and Córdoba, in the central region of Argentina. In these provinces, the animals sampled were mainly dairy cows (mainly Holstein and Jersey cows). In this region, numerous severe outbreaks of *T*. *vivax* infection occurred in 2017, and the disease remains endemic in this area [17]. Six areas were strategically selected from the regions with significant livestock populations. The geographical locations and names of the six surveyed areas, all situated within the central region, are shown on a map (Fig 1). Maps were generated using the Python 3.10 programming language (https://www.python.org/downloads/release/python-3100/); shape files are freely available at https://www.ign.gob.ar/NuestrasActividades/InformacionGeoespacial/CapasSIG. Infected animals were routinely treated with diminazene aceturate. Blood samples were taken between January 2021 and January 2023. Approximately 5 mL of blood was drawn from the jugular vein and placed in plastic tubes for serum preparation to ensure room temperature conditions. Serum samples were then stored at −20°C. A total of 892 blood samples were randomly collected from cattle aver a two-years period (2021–2023) from a total of 40 small farms. These samples were then tested using the microhematocrit technique (MHT), as previously described [33], within 3–6 h of blood collection. Each animal was sampled only once during the course of the study, using a cross-sectional approach. Microscopic analysis of Giemsa-stained blood smears from the affected animals was performed to assess the presence of bovine babesiosis and anaplasmosis.

### ELISA assay

The indirect ELISA was performed according to the current protocol. Microtiter plates (Microlon High Binding, Greiner) were coated with 500 ng of specific antigens (TvISGAf and/or TvISGAm) in 50 mM carbonate-bicarbonate buffer (pH 9.6) and incubated overnight at 4°C. The plates were washed thrice with 0.05% Tween 20 in phosphate-buffered saline (PBS-T) and then blocked with PBS containing 5% skimmed milk. Following another wash with PBS-T, serum samples were added to each well (diluted 40 times in 1% skimmed milk in PBS) and incubated at 37°C for 1 h. A subsequent round of washing was performed, and peroxidase-conjugated goat anti-bovine IgG (diluted 10 000 times in 1% skimmed milk in PBS, Sigma-Aldrich) was added and incubated at 37°C for 1 h. For substrate application, 3, 3’, 5, 5’;-tetramethylbenzidine (Invitrogen) was used, and the reaction was halted by the addition of 1 M sulfuric acid. The optical density (OD) was measured at 450 nm using an ELx808 microplate reader (BioTek). For each specific antigen, the results were presented as the average OD derived from two concurrent evaluations of the same serum sample. Within each plate and for each specific antigen, six negative controls (seronegative for *T*. *vivax*) were examined simultaneously. The cut-off values for the ELISA results were determined by calculating the mean OD of the negative control serum samples, in addition to two standard deviations. Antibody levels were quantified as the ratio between the OD of the sample and the cut-off value. This measure is referred to as IODN (index of the optical density of the antibodies relative to the negative control) [35]. The IODN value below which it was considered negative was determined using ROC curve analysis.

### Data analysis

A Kolmogorov-Smirnov test was initially conducted to assess the normality of the samples prior to comparing positive and negative serum outcomes. If the samples exhibited a normal distribution, comparisons between groups were performed using unpaired Student’s t-test. For samples that did not display a normal distribution, the Mann-Whitney U test was used for comparisons. Statistical significance was set at *P* ≤ 0.05. The performance of each assay was assessed using sensitivity (Se), specificity (Sp), positive predictive value (PPV), negative predictive value (NPV), area under the curve (AUC), and accuracy (AC). ROC analysis was employed to determine the lower limit of positivity (cutoff) and ascertain the optimal combination of sensitivity, specificity, and AUC. The quality of each test was classified based on the AUC results from the ROC analysis as follows: "Excellent" (1.0–0.9), "Good" (0.9–0.8), "Reasonable" (0.8–0.7), "Poor" (0.7–0.6), and "Fail" (< 0.6). To assess the relationship between variables (time vs. antibody levels (IODN)) within each group, Pearson’s correlation test was performed. Subsequently, a general linear model was applied to each group using regression analysis. For statistical analysis, GraphPad Prism 7.0 and MedCalc 12.2.1. software were used.

## Results

### *In silico* analysis of sequence encoding for a *T*. *vivax* invariant surface glycoprotein and its recombinant expression

The information available in the database of the *T*. *vivax* Y486 genome project database (http://tritrypdb.org/tritrypdb/) contains a 1203 bp open reading frame (TvY486_0045500) that encodes an invariant surface glycoprotein of 400 amino acids with a theoretical molecular mass of 44.5 kDa, S1 Fig, previously analyzed by Fleming et al., 2016 [24]. Based on this information, we searched the genomic data of an American *T*. *vivax* isolate [29] for a putative gene encoding an invariant surface glycoprotein. Using the BLASTn tool and the nucleotide sequence of TvY486_0045500, we identified a nucleotide sequence encoding a putative invariant surface glycoprotein (tig00000163, [29]). The amino acid sequence is presented in S2 Fig. *In silico* analysis predicted that both African and American *T*. *vivax* invariant surface glycoproteins have a typical ISG domain structure with an N-terminal signal peptide, a transmembrane domain, and an intracellular domain (S1 and S2 Figs). Amino acid sequence analysis of African and American *T*. *vivax* ISG revealed that these proteins share ~63% identity and ~69% similarity (S3 Fig).

**Fig 2:**
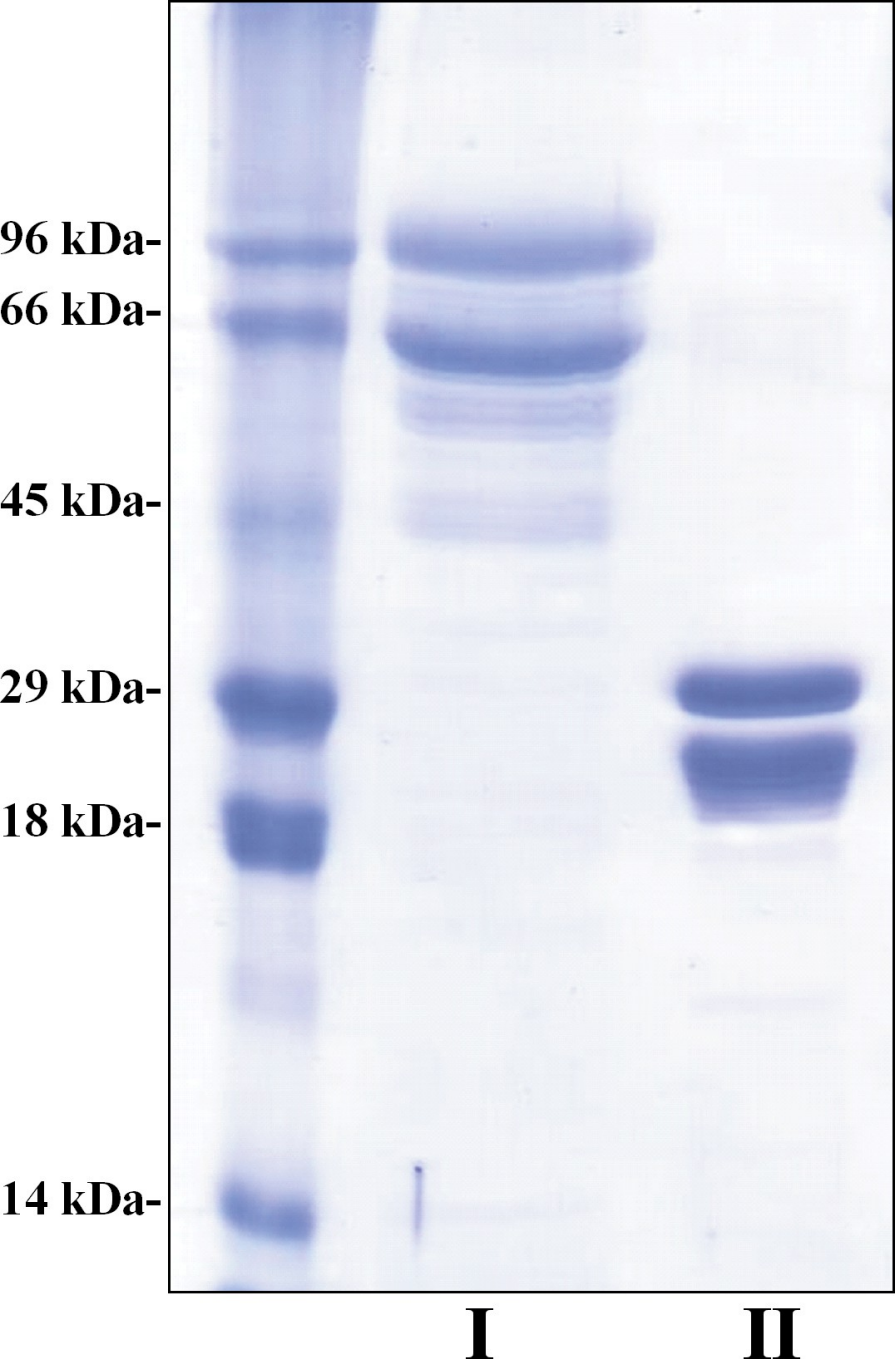
SDS-PAGE of purified recombinant proteins. Purified recombinant proteins were defined by electrophoretic migration under reducing and denaturing conditions followed by Coomassie blue staining. **MW**; **Lane I**: TvISGAf (1 μg); **Lane II**: TvISGAm (1 μg).

Based on previous information [24] and predictions of linear epitopes (S4 Fig), we amplified truncated ISG sequences from African (TvISGAf) and American (TvISGAm) *T*. *vivax* genomic DNA by PCR (without the signal peptide and N-terminal transmembrane domain). The resulting DNA sequences were cloned into the pGEM-T Easy vector for analysis. To characterize the antigenic functionality of the recombinant proteins, we first tried sought to express the recombinant antigens (TvISGAf, from amino acid 126 to 400 and TvISGAm, from amino acid 125 to 274) in *E*. *coli* as His-tag or N-terminal MBP His-tag fusion proteins using the plasmids pET28 and pMOMAL, respectively. After evaluating the different induction conditions, we were only able to obtain the TvISGAm antigen as an N- and C-terminal double His-tag fusion protein and the TvISGAf as a fusion protein with an N-terminal MBP His-tag. These expression systems allowed us to successfully obtain the overexpression of the recombinant antigens in soluble form. Therefore, we continued the serological evaluations using the recombinant antigens obtained by these two strategies. Similar difficulties with the recombinant expression of the antigen derived from the African strain have been described previously [24]. The amino acid sequences of the recombinant proteins are shown in S5 Fig. SDS-PAGE analysis showed that the recombinant proteins were produced and isolated with high electrophoretic purity (Fig 2). However, a band of lower apparent molecular mass with similar relative abundance was observed in both cases (Fig 2). Co-purification of a truncated product could explain this result, since the purification tags are located towards the N-terminus of the recombinant proteins. The purified recombinant proteins were stored at -80°C for at least 8 months.

**Fig 3:**
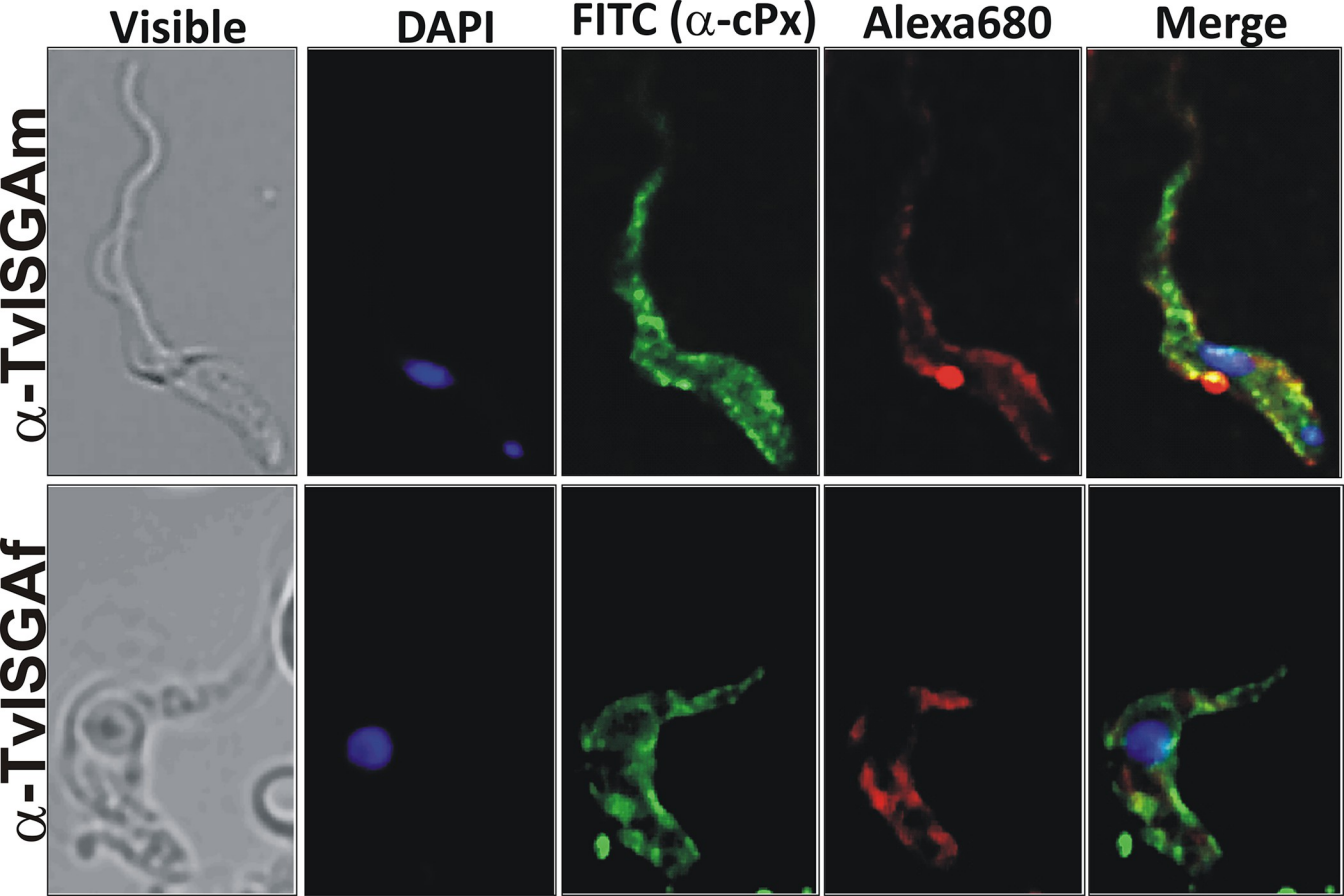
Immunodetection of ISG proteins in *T*. *vivax*. Confocal microscopy images were obteined by the co-immunolocalization of ISG (labeled with mouse anti-TvISGAf or anti-TvISGAm antibody and Alexa680 conjugated goat anti-IgG as a secondary antibody, red) and cytoplasmic peroxiredoxin (cPx, labeled with rabbit anti-cPx antibody and FITC-conjugated goat anti-IgG as a secondary antibody, green). DAPI was used for the nuclear and kinetoplast staining (blue). Negative controls (incubations without primary antibodies) are shown in the S6 Fig.

As mentioned above, the SOSUI prediction server suggested that the ISG protein is a transmembrane protein. Using mouse-specific antibodies against recombinant TvISGAf and TvISGAm, we investigated the localization of the ISG protein in African *T*. *vivax* cells. Using immunofluorescence microscopy (Fig 3), we detected recognition signals throughout the parasite plasma membrane. This is in contrast to the signal observed for cytoplasmic peroxiredoxin, which presents a recognition signal throughout the cell (Fig 3). These results support a cell surface localization of ISG in *T*. *vivax* cells.

### Preliminary antigen evaluation

The purified recombinant trypanosome proteins were used to prepare ELISA plates as described in the Experimental Procedures section, and assayed against 60 samples from the sera panel, divided into cattle infected with *T*. *vivax* (n = 30) and uninfected cattle (n = 30). Coated ELISA plates were evaluated using all individual sera. These antigens, including TvISGAf (TvISGAf-based ELISA), TvISGAm (TvISGAm-based ELISA), and a combination of both proteins (TvISG-based ELISA), were evaluated. Different mixing conditions were evaluated for the proportion of each protein in the mixture. It was found that a 1:1 ratio provided optimal discrimination. No cross-reactivity to the MBP-tagged proteins was observed in the evaluated positive and negative sera (S7A Fig).

**Fig 4:**
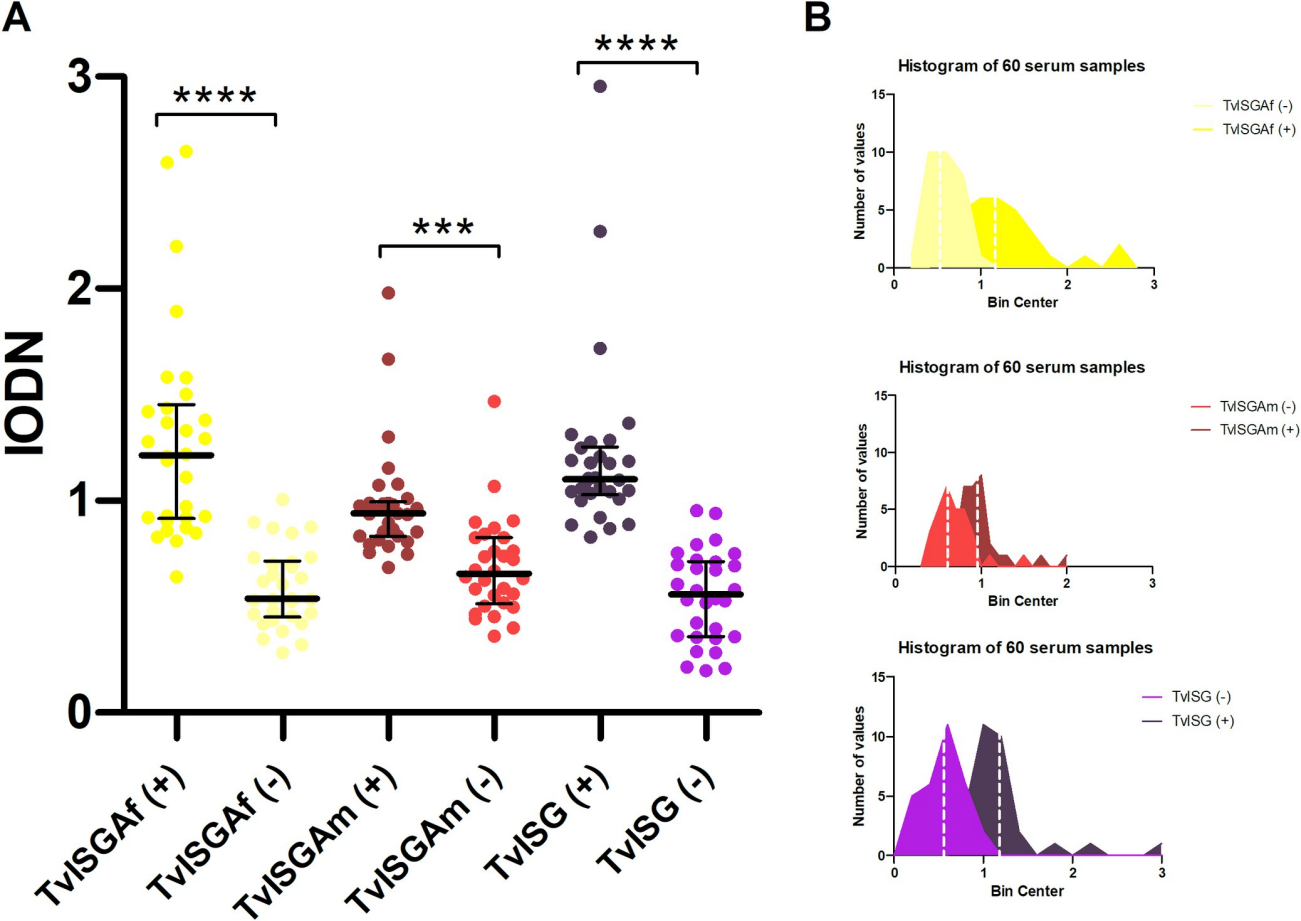
Comparison reactivity of TvISGAf (yellow) or TvISGAm (orange) antigens or their mixture (TvISG, purple) in ELISA using confirmed positive and negative results from dairy cattle sera (A). Histograms shown the distribution of the populations of positive and negative sera (B). *** *p* < 0.001, **** *p* < 0.0001.

The data are presented as box plots for each individual recombinant antigen ELISA plate (Fig 4), allowing visualization of the IODN range. The data showed that both ELISA assays, one based on TvISGAf and the other on TvISG (a combination of TvISGAf and TvISGAm), demonstrated significant discrimination between positive and negative samples. The ELISA based on TvISGAm alone showed a significant overlap between the populations of positive and negative sera, as observed in the histogram (Fig 4B). However, the detection of antibodies using TvISGAm antigen in the TvISG-based ELISA was improved when combined with TvISGAf. Therefore, we decided to evaluate the complete panel of sera using only the TvISGAf-based and TvISG-based ELISA.

In addition, the antigens evaluated in the ELISA gave negative results (below the positivity threshold) when tested with bovine sera from animals infected with *Anaplasma marginale*, *Babesia bovis*, and *T*. *theileri* (S7B Fig).

### Sensitivity and specificity of TvISGAf-based ELISA and TvISG-based ELISA

**Fig 5:**
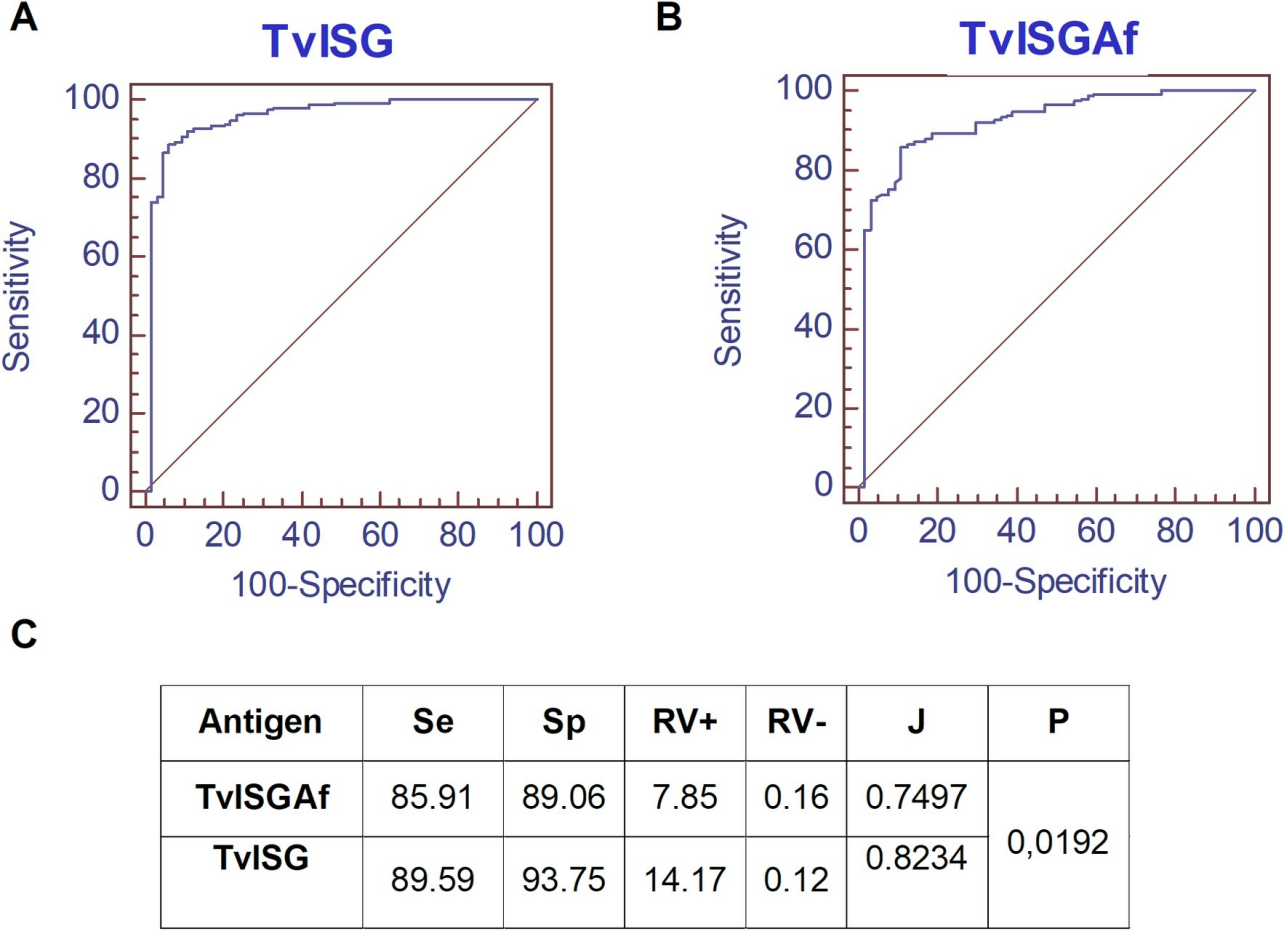
Comparison of the ROC curves of the anti-IgG ELISA tests of bovine sera using TvISGAf antigen and TvISG mixture. ROC curves for the TvISG mixture of TvISGAf and TvISGAm antigens (A) and TvISGAf (B). Different ELISA assays were performed using sera from the following groups: negative control (n = 63) and animals positive for *T*. *vivax* by parasitology and PCR tests (n = 149). Table of diagnostic performance (C); Se, sensitivity; Sp, specificity; RV, likelihood ratio; J, Youden index; AUC, area under the ROC curve; P, pairwise comparison of ROC curves.

The formal parameters of sensitivity and specificity (proportion of correct negative results) for each test were determined by ROC curve analysis using 212 serum samples (Fig 5). The combination of the two ISGs in the TvISG-based ELISA showed the most significant discrimination between *T*. *vivax*-infected cattle and control cattle, with an area under the ROC curve of 0.951, whereas that of the TvISGAf-based ELISA was 0.921. The TvISG-based ELISA using the antigen mixture achieved a sensitivity of 89.6% (95% CI of 82.4% to 93.2%) and a specificity of 93.8% (95% CI of 84.8% to 98.3%), whereas the sensitivity and specificity of the TvISGAf-based ELISA were 85.9% (95% CI of 79.1% to 91.1%) and 89.1% (95% CI of 78.8% to 95.5%), respectively. In addition, pairwise comparison of the ROC curves showed significant differences between the mixture and TvISGAf (*p* = 0.0192).

### Seroprevalence of bovine trypanosomosis in the central region of Argentina by TvISG-based ELISA

In this study, 892 samples collected from dairy cows in 40 fields in the central region of Argentina were tested using the TvISG-based ELISA (Fig 1). The seroprevalence for bovine trypanosomosis was determined and is presented in Table 1. *T*. *vivax* infection was detected in 472 of 892 (53%) samples. Notably, the highest seroprevalence for *T*. *vivax* was observed in San Justo, Córdoba province, and San Cristobal, Santa Fe province, with rates of 69% and 68%, respectively. In other regions, seroprevalence in the different departments of Santa Fe province was 55% in San Martin, 50% in Castellanos, 42% in San Jeronimo, and 21% in Vera. Overrall, the province of Santa Fe had an overall seroprevalence rate of 47%.

**Table 1:** Seroprevalence of *T*. *vivax* among cattle (n = 892) in two provinces in Argentina was determined using TvISG-based ELISA.

Province	IN	Department	Location	Number of serum samples	TvISG-based ELISA	
					Number of positive samples	Seroprevalence (%)
Santa Fe	1	Vera	La Gallareta	139	29	21
		San Cristobal	Suardi San Guillermo	84 30	60 18	68
	2					

	3	Castellanos	Humberto Primo Estación Clucellas Colonia Bicha	90 60 24	26 44 17	50
	4	San Martin	Colonia Belgrano Carlos Pellegrini	132 40	78 16	55
	5	San Jerónimo	San Genaro Norte	67	28	42
Córdoba	6	San Justo	Morteros San Francisco Colonia Iturraspe Colonia Silva Colonia San Pedro	80 34 30 31 51	44 12 27 26 47	69
Total				892	472	53

Serum samples were collected during the period 2021–2023.

IN: Identification number from each Department mentioned in Fig 1.

## Discussion

Bovine trypanosomosis caused by *T*. *vivax* is currently diagnosed by a combination of clinical, parasitological, molecular, and serological techniques [36–38]. Parasitological diagnosis is the most commonly used method to identify *T*. *vivax* in the field, although its low sensitivity is only suitable for diagnosis during a short period of high parasitemia [39]. The most commonly used diagnostic tests to determine the prevalence of trypanosomosis are IFAT and ELISA. However, the IFAT requires a host for parasite culture, which making it less scalable and more challenging to standardize [19]. Crude extracts have been used to diagnose both *T*. *vivax* and *T*. *evansi* in the acute and chronic phases of the disease [40]. Given the cross-reactivity observed with extracts of both parasites [19,41], recombinant antigens have been proposed for the specific serodiagnosis of *T*. *vivax* [6,36].

Several antigens have been proposed for the diagnosis of bovine trypanosomosis [20–24]. Fleming et al. (2016) identified two ISGs (TvY486_0019690 and TvY486_0045500) from an African strain of *T*. *vivax*, with TvY486_0045500 demonstrating the most promising diagnostic performance [24]. In this study, we evaluated a truncated variant of the TvY486_0045500 antigen (TvISGAf) against a panel of 212 serum samples from Argentina. The results of the diagnostic performance of TvY486_0045500 reported by Fleming et al. (2016) demonstrated a sensitivity and specificity of 94.5% and 88.0%, respectively [24]. However, our ELISA using the same antigen demonstrated comparable specificity but a 10% reduction in sensitivity. We used a smaller fraction, omitting the non-globular region of the protein that was attached to the anchor site and did not present immunodominant B epitopes. This discrepancy may be attributed to the fact that the sera evaluated by Fleming et al. (2016) were derived from African cattle, whereas the sera in our study were from Latin American cattle [24]. Furthermore, the antigen cloned by Fleming et al. (2016) and reproduced by us as TvISGAf is derived from the DNA of an African strain, Y468 [24]. The infecting strains in Latin America are phylogenetically distinct from African strains [42,43], which could contribute to the observed decrease in sensitivity.

To improve diagnostic performance, we evaluated an ISG candidate similar to TvY486_0045500 from a South American isolate (referred to as IB, [29]). TvISGAm exhibited approximately 63% identity and 69% similarity with TvISGAf. Furthermore, by evaluating the localization of these ISGs, we demonstrated that the antibodies generated by the evaluated recombinant ISGs recognized the *T*. *vivax* membrane (Fig 3). However, when we evaluated the diagnostic capacity, TvISGAm alone did not yield favorable results. This reduced reactivity may be due to the fact that although both TvISGAf and TvISGAm antigens have a high degree of similarity, TvISGAm contains different epitope sequences that are less recognized by the bovine immune system. However, some sera have shown recognition of TvISGAm in contrast to TvISGAf. Consequently, the combined performance of these antigens in the detection of anti-*T*. *vivax* IgG in bovine serum demonstrated an increase in the Youden index of 0.074 and a statistically significant improvement in the area under the curve (p = 0.0192), indicating an increased sensitivity (over 3.7%) and specificity (over 4.7%) compared to the performance obtained for ELISA based on TvISGAf.

Finally, we conducted a field seroprevalence study using a TvISG-based ELISA in dairy cattle, in the central provinces of Argentina. The study included 40 farms located in the dairy basin of the country, with particular emphasis on the provinces of Santa Fe and Córdoba. It is noteworthy that within the same geographical region, Florentin et al. (2021) performed parasite detection of *T*. *vivax* infection in cattle using PCR and reported a positivity rate ranging from 39% to 55% [17]. Remarkably, the overall seroprevalence of 53% documented in our study is in close agreement with the molecular diagnostic results obtained four years earlier. Although molecular and serologic detection methods are not directly comparable, these two independent studies provide critical evidence of the substantial presence of *T*. *vivax* in livestock in the region. Furthermore, our results reveal a notable discrepancy in prevalence between the provinces of Córdoba and Santa Fe, with Córdoba having a seroprevalence that is almost 20% higher than that of Santa Fe. This discrepancy may be attributed to the distribution of dairy farms in these provinces. While cases in Santa Fe were collected from farms distributed across a larger area, yielding rates ranging from 21% to 68%, only five farms in the department of San Justo were sampled in Córdoba. It is noteworthy that both Córdoba and Santa Fe are endemic regions for *T*. *vivax* and together account for almost 70% of Argentina’s milk production [17].

Our findings are consistent with those of other studies conducted in South America, indicating a high prevalence of this disease in endemic regions. An epidemiological study in the Pantanal region of Brazil emploing indirect ELISA reported an average seroprevalence rate of 56% [41]. Moderate seroprevalence of *T*. *vivax* has been documented in other Latin American countries. In Peru, the prevalence was 14%, in Ecuador it was 23%, in Paraguay it was 40% [44], and in Bolivia it was 27% [45]. The 53% prevalence rate reported in Argentina indicates that the parasite continues to spread throughout South America. Given the lack of control and surveillance mechanisms, it is possible that the infection may invade other countries outside Africa and Latin America [1]. This is evidenced by the first Asian case, which was detected in Iran [46].

Although *T*. *vivax* was initially documented in Argentina in 2006 [15], it was not until the summers of 2016 and 2017 that the first outbreaks were recorded in dairy farms [47]. It is crucial to acknowledge that the clinical manifestations of bovine trypanosomosis caused by *T*. *vivax* are non-specific, rendering clinical diagnosis challenging and imprecise, particularly in the presence of other cattle diseases such as anaplasmosis, babesiosis, bovine leptospirosis, leukemia, neosporosis, and viral infections [14,37,48]. The lack of awareness of the disease, the large number of registered dairy farms in the dairy area [49], the sale of infected animals, and the use of the same syringes for vaccination have contributed to its rapid spread between the provinces of Córdoba and Santa Fe. Significant economic impacts have been caused by losses in dairy cattle infected with *T*. *vivax*, leading to the treatment of entire herds and resulting in a substantial reduction in milk production from affected herds [17,47]. In this study, we demonstrated high seroprevalence of the disease in an affected region, suggesting a possible spread of the disease in the southern and eastern areas of the Pampas region, as observed by other authors [17,50].

The control of bovine trypanosomosis is a significant concern in countries affected by the disease due to the significant economic losses incurred. Currently, no vaccine is available for this disease, and climate change is conducive to the spread of vector insects to increasingly less temperate areas [51]. Therefore, precise detection and control programs are necessary. The results of this study suggest the need for bovine trypanosomosis surveillance programs not only in endemic regions, but also in other countries with intensive livestock production. The sensitivity and specificity of the TvISG-based ELISA, designed to detect specific antibodies against *T*. *vivax*, render it a valuable addition to surveillance programs.

## Supporting information

S1 Fig Transmembrane domain prediction of TvY486_0045500 protein. The transmembrane domain probability profile was generated using SOSUI (http://harrier.nagahama-i-bio.ac.jp/sosui/).
(PDF)

S2 Fig Transmembrane domain prediction of Invariant Surface Glycoprotein from American *T*. *vivax*. The transmembrane domain probability profile was generated using SOSUI (http://harrier.nagahama-i-bio.ac.jp/sosui/).
(PDF)

S3 Fig Amino acid sequence alignment of Invariant Surface Glycoprotein from African and American *T*. *vivax*. Amino acid alignment was constructed using the Clustal W algorithm.
(PDF)

S4 Fig Prediction of linear antigenic epitopes of Invariant Surface Glycoprotein from African and American *T*. *vivax*. The prediction probability profile was generated using SVMTriP (http://sysbio.unl.edu/SVMTriP/index.php).
(PDF)

S5 Fig Full amino acid sequences of recombinant TvISGAf and TvISGAm proteins. The sequences of the fusion-tags are highlighted in green, and the *T*. *vivax* proteins are indicated in yellow.
(PDF)

S6 Fig Negative control of immunodetection of ISG protein in *T*. *vivax* cells. Fluorescence microscopy images were performed without primary antibodies (mouse anti-TvISGAf or mouse anti-TvISGAm or rabbit anti-cPx) in the presence of Alexa680 conjugated goat anti-IgG and FITC conjugated goat anti-IgG. DAPI was used for nuclear and kinetoplast staining (blue).
(PDF)

S7 Fig Evaluation of cross-reactivity in TvISG ELISA. **a)** Cross-reactivity with the fusion protein MBP. **b)** Cross-reactivity with samples from cattle infected with Babesia bovis (20), Anaplasma marginale (20), and Trypanosoma theileri (8). Cutoff values of the tests were 0.80 (indicated by broken lines).
(PDF)

S1 Table Primers used for amplifying the encoding sequences of Invariant Surface Glycoprotein from African and American *T*. *vivax*. (PDF)
