# Supplementary material for: Improved serodiagnosis of Trypanosoma vivax infections in cattle reveals high infection rates in the livestock regions of Argentina
Source: PLoS Negl Trop Dis. 2024 Jun 26;18(6):e0012020. doi: 10.1371/journal.pntd.0012020 (PMC11233006; doi:10.1371/journal.pntd.0012020)
Supplement: S1 Fig — The transmembrane domain probability profile was generated using SOSUI (http://harrier.nagahama-i-bio.ac.jp/sosui/). (PDF) [file pntd.0012020.s001.pdf]

>TvY486_0045500 – Invariant Surface Glycoprotein (*T. vivax* Y486)

**MRAQKRSDHVLLNMQMRRYGPPLFVAALCLIDVVLCQASSYENEIARALCKMGSTHRRMSMVFGVLQQRISK
TDDTINGLETDLWKLKKAGLPDEKYQEVNDKVINVTGSVSLVTNAVKVAQKKLEEFIEKVKTEHYNDHYLKL
EDRKFGESVSNCRDWATYNEETPDKLRKKLESGLKTLEAWATEESNEWEKEQKEVESDLLSKENRNSLQYGT
LHTAFKDLVKSMMVELTTVSFYMPKALEGVPGADAAVNEARKFVVVAMANECQSVASEAAASEEKQAQCEK
LNKKLQEIKEKKRQAIGGDSEGPKSSDAKSTDATPTSSASQKVIVEEVLDSADGDELMELVQTADKPSAANNSK
LSPTNLALAISIPVALVLIGAAVFLVMRRRTAEKVVPTI**

Result of SOSUI prediction

TvY486_0045500 is MEMBRANE protein

No.	region	transmembrane seq.	type
1	19-41	YGPPLFVAALCLIDVVLCQASSY	Primary

Hydropathy & Charge plot

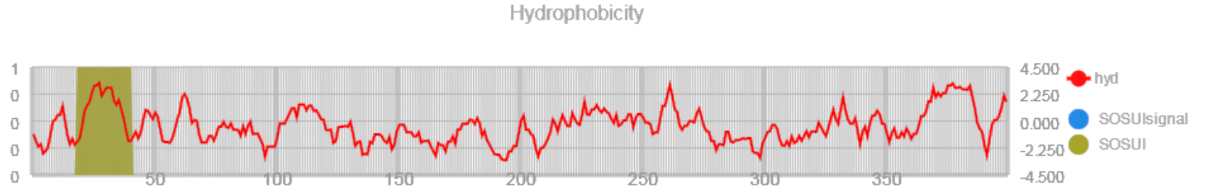

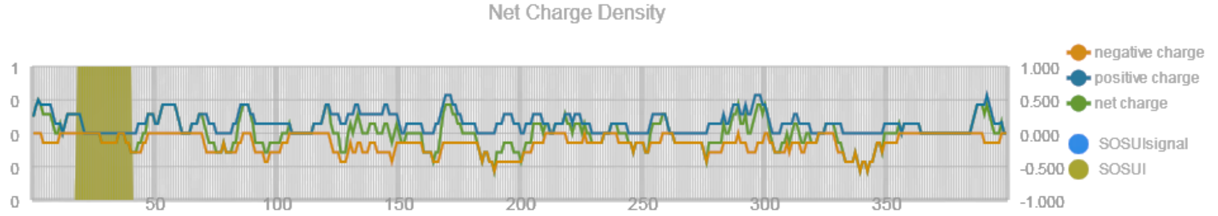

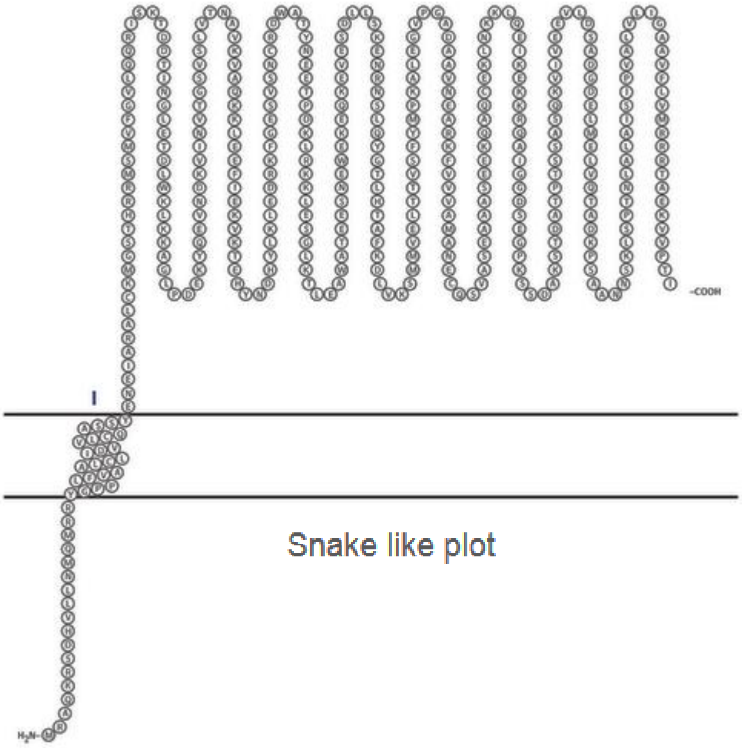

Snake like plot

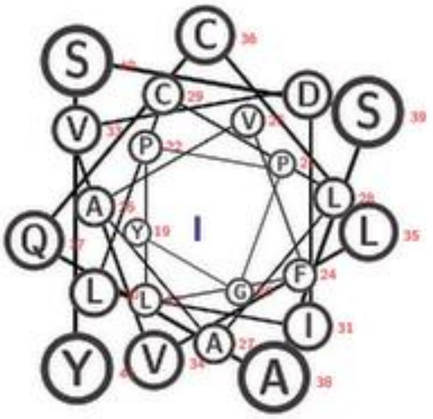

Wheel plot of transmembrane helices

S1 Fig: Transmembrane domain prediction of TvY486_0045500 protein. The transmembrane domain probability profile was generated using SOSUI (http://harrier.nagahama-i-bio.ac.jp/sosui/).
