# Supplementary material for: Improved serodiagnosis of Trypanosoma vivax infections in cattle reveals high infection rates in the livestock regions of Argentina
Source: PLoS Negl Trop Dis. 2024 Jun 26;18(6):e0012020. doi: 10.1371/journal.pntd.0012020 (PMC11233006; doi:10.1371/journal.pntd.0012020)
Supplement: S2 Fig — The transmembrane domain probability profile was generated using SOSUI (http://harrier.nagahama-i-bio.ac.jp/sosui/). (PDF) [file pntd.0012020.s002.pdf]

> tig00000163 – Invariant Surface Glycoprotein from American *T. vivax*

**MGEHKRSDHVLLNMQVRRYVVPLFVVALCSLDVVRCHARSYENEIARALCKMGTTHRRMDMVFGVLKQNIS
NTEGTMIELETDLWKLWKAGLPDEKYKEVDKSQRRGKQHLLRDKRCEGCPEKLEEFIEKVKTEHYNDHYLK
LEDRKFGESVSNCRDWATYNEETPEQLRKSSSRALKPSKPGPRKSQGSGKRRKXGGIRFAKRRKQXSIRVRNS
AHGFKDLVESLMVKLKTACTYLPKTLEGVPGAEVAVNEARKFVVVAMANECQSVASDAAASEEKHEKCEKL
NKKLQEIKEKKQQANNGDSEGPKSSDAKSADATPTSSASQKVIVEEVLDSADGDELMELVQTADKPSSANNSKL
TPPTWRSPFPSRLLLFSSVLLRSWXCAARRKRLSPQY**

Result of SOSUI prediction

TvISGAM is MEMBRANE protein

No.	region	transmembrane seq.	type
1	19-41	YVVPLFVVALCSLDVVRCHARSY	Primary

Hydropathy & Charge plot

Hydrophobicity

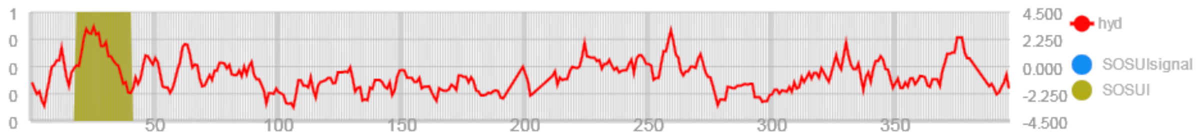

Net Charge Density

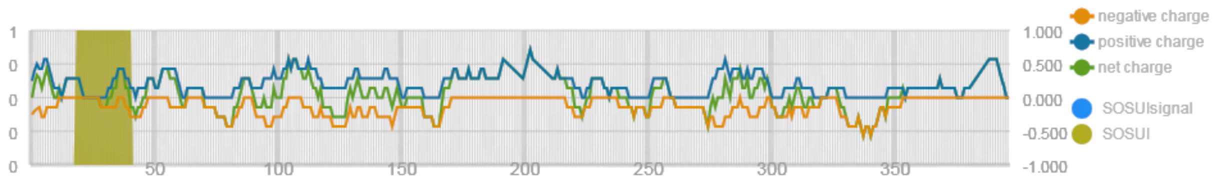

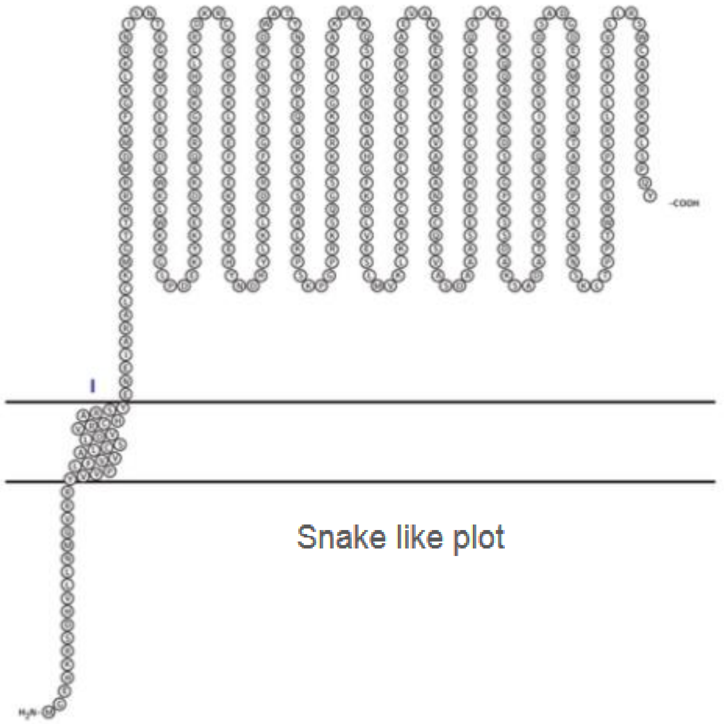

Snake like plot

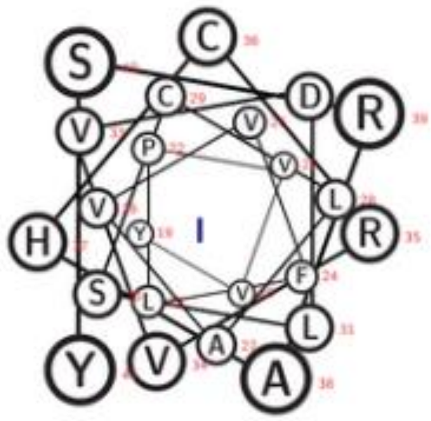

Wheel plot of transmembrane helices

<u>S2 Fig:</u> Transmembrane domain prediction of Invariant Surface Glycoprotein from American *T. vivax*. The transmembrane domain probability profile was generated using SOSUI (http://harrier.nagahama-i-bio.ac.jp/sosui/).
