# Supplementary material for: Improved serodiagnosis of Trypanosoma vivax infections in cattle reveals high infection rates in the livestock regions of Argentina
Source: PLoS Negl Trop Dis. 2024 Jun 26;18(6):e0012020. doi: 10.1371/journal.pntd.0012020 (PMC11233006; doi:10.1371/journal.pntd.0012020)
Supplement: S3 Fig — Amino acid alignment was constructed using the Clustal W algorithm. (PDF) [file pntd.0012020.s003.pdf]

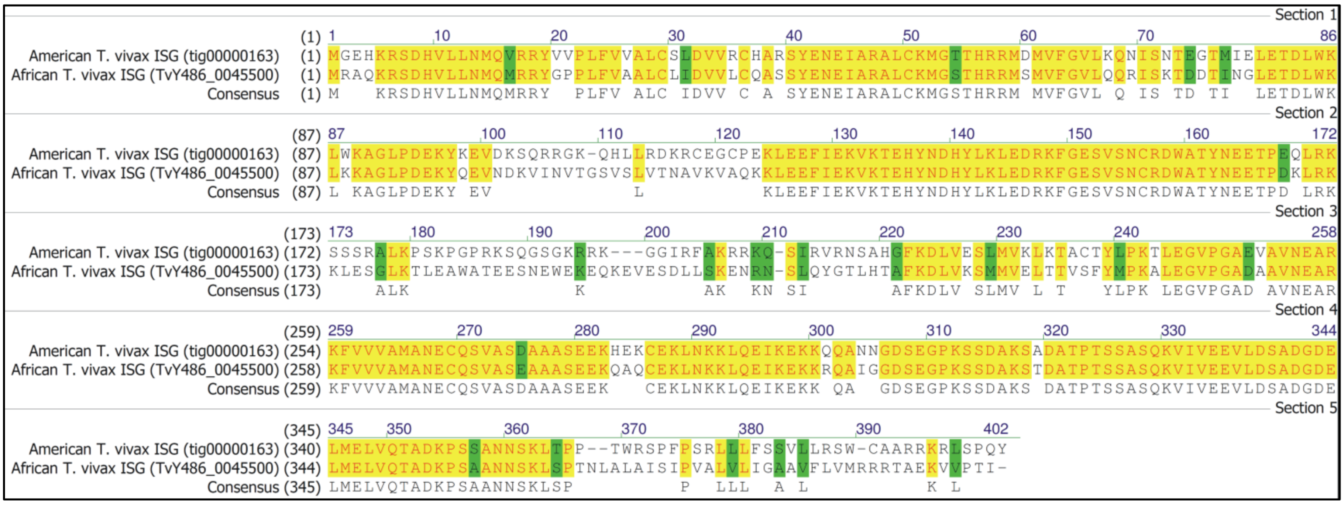

<u>S3 Fig:</u> Amino acid sequence alignment of Invariant Surface Glycoprotein from African and American *T. vivax*. Amino acid alignment was constructed using the Clustal W algorithm.
