# Supplementary material for: Improved serodiagnosis of Trypanosoma vivax infections in cattle reveals high infection rates in the livestock regions of Argentina
Source: PLoS Negl Trop Dis. 2024 Jun 26;18(6):e0012020. doi: 10.1371/journal.pntd.0012020 (PMC11233006; doi:10.1371/journal.pntd.0012020)
Supplement: S4 Fig — The prediction probability profile was generated using SVMTriP (http://sysbio.unl.edu/SVMTriP/index.php). (PDF) [file pntd.0012020.s004.pdf]

>TvY486_0045500 – Invariant Surface Glycoprotein (*T. vivax* Y486)

Epitope predicted inside protein below:

Rank	Location	Epitope	Score	Recommend*
1	214 - 233	YGTLHTAFKDLVKSMMVELT	1.000	●
2	39 - 58	SSYENEIARALCKMGSTHRR	0.823	●
3	333 - 352	EEVLDSADGDELMELVQTAD	0.729	
4	107 - 126	VTGSVSLVTNAVKVAQKKLE	0.701	
5	253 - 272	VNEARKFVVVAMANECQSVA	0.509	
6	139 - 158	DHYLKLEDRKFGESVSNCRD	0.399	
7	178 - 197	LKTLEAWATEESNEWEKEQK	0.306	
8	381 - 400	GAAVFLVMRRRTAEKVVPTI	0.297	
9	303 - 322	IGGDSEGPKSSDAKSTDATP	0.289	

>tig00000163 – Invariant Surface Glycoprotein from American *T. vivax*

Epitope predicted inside protein below:

Rank	Location	Epitope	Score	Recommend*
1	39 - 58	RSYENEIARALCKMGTTHRR	1.000	●
2	213 - 232	SAHGFKDLVESLMVKLKTAC	0.996	●
3	244 - 263	GAEVAVNEARKFVVVAMANE	0.983	●
4	329 - 348	EEVLDSADGDELMELVQTAD	0.940	
5	138 - 157	DHYLKLEDRKFGESVSNCRD	0.514	
6	95 - 114	EKYKEVDKSQRRGKQHLLRD	0.492	

S4 Fig: Prediction of linear antigenic epitopes of Invariant Surface Glycoprotein from African and American *T. vivax*. The prediction probability profile was generated using SVMTriP (http://sysbio.unl.edu/SVMTriP/index.php).
