# Supplementary material for: Improved serodiagnosis of Trypanosoma vivax infections in cattle reveals high infection rates in the livestock regions of Argentina
Source: PLoS Negl Trop Dis. 2024 Jun 26;18(6):e0012020. doi: 10.1371/journal.pntd.0012020 (PMC11233006; doi:10.1371/journal.pntd.0012020)
Supplement: S5 Fig — The sequences of the fusion-tags are highlighted in green, and the T. vivax proteins are indicated in yellow. (PDF) [file pntd.0012020.s005.pdf]

>TvISGAf (MBP6His-fusion protein of 958 amino acid and 106.4 kDa)

**MKIEEGKLVIWINGDKGYNGLAEVGKKFEKDTGIKVTVEHPDKLEEKFPQVAATGDGPDIIFWAHD
RFGGYAQSGLLAEITPDKAFQDKLYPFTWDAVRYNGKLIAYPIAVEALSLIYNKDLLPNPPKTWEEIP
ALDKELKAKGKSALMFNLQEPYFTWPLIAADGGYAFKYENGKYDIKDVGVDNAGAKAGLTFLVDL
IKNKHMNADTDYSIAEAAFNKGETAMTINGPWAWSNIDTSKVNYGVTVLPTFKGQPSKPFVGVLSA
GINAASPNKELAKEFLENYLLTDEGLEAVNKDKPLGAVALKSYEEELAKDPRIAATMENAQKGEIM
PNIPQMSAFWYAVRTAVINAASGRQTVDEALKDAQTNSSSNNNNNNNNNNLGIEGRISHHHHHHEN
LYVNEFIEKVKTEHYNDHYLKLEDRKFGESVSNCRDWATYNEETPDKLRKKLESGLKTLEAWATE
ESNEWEKEQKEVESDLLSKENRNSLQYGTLHTAFKDLVKSMMVELTTVSFYMPKALEGVPGADAA
VNEARKFVVVAMANECQSVASEAAASEEKQAQCEKLNKKLQEIKEKKRQAIGGDSEGPKSSDAKST
DATPTSSASQKVIVEEVLDSADGDELMELVQTADKPSAANNSKLSPTNLALAISIPVALVLIGAAVFLV
MRRRTAEKVVPTI**

>TvISGAm (double His-tag-fusion protein of 190 amino acid and 21.4 kDa)

**MGSSHHHHHHSSGLVPRGSHMASGSEFIEKVKTEHYNDHYLKLEDRKFGESVSNCRDWATYNEET
PEQLRKKLESGLETLEAWATEESGKWEKEKNEVESDLLKDENNKASEYATLRTAFKDLVESLMVK
LKTACTYLPKTLEGVPGAEVAVNEARKFVVVVMANECQSVASDAAGKLAAALEHHHHHH**

S5 Fig: Full amino acid sequences of recombinant TvISGAf and TvISGAm proteins. The sequences of the fusion-tags are highlighted in green, and the *T. vivax* proteins are indicated in yellow.
