# Supplementary material for: Improved serodiagnosis of Trypanosoma vivax infections in cattle reveals high infection rates in the livestock regions of Argentina
Source: PLoS Negl Trop Dis. 2024 Jun 26;18(6):e0012020. doi: 10.1371/journal.pntd.0012020 (PMC11233006; doi:10.1371/journal.pntd.0012020)
Supplement: S6 Fig — Fluorescence microscopy images were performed without primary antibodies (mouse anti-TvISGAf or mouse anti-TvISGAm or rabbit anti-cPx) in the presence of Alexa680 conjugated goat anti-IgG and FITC conjugated goat anti-IgG. DAPI was used for nuclear and kinetoplast staining (blue). (PDF) [file pntd.0012020.s006.pdf]

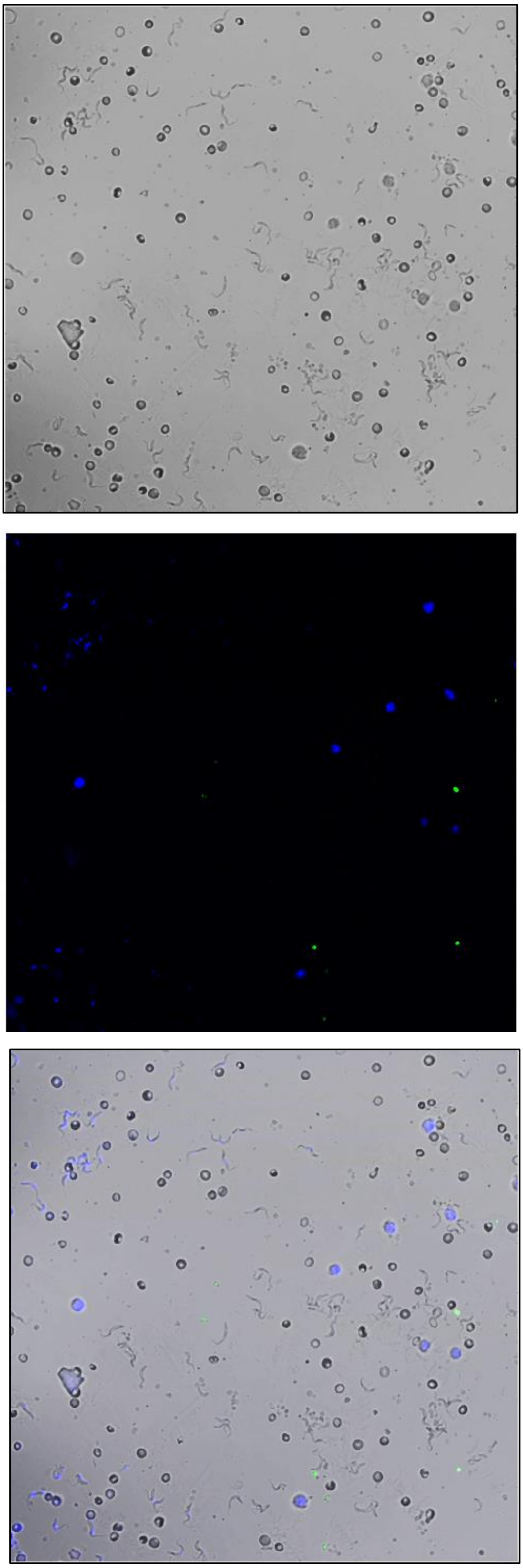

<u>S6 Fig:</u> Negative control of immunodetection of ISG protein in *T. vivax* cells. Fluorescence microscopy images were performed without primary antibodies (mouse anti-TvISGAf or mouse anti-TvISGAm or rabbit anti-cPx) in the presence of Alexa680 conjugated goat anti-IgG and FITC conjugated goat anti-IgG. DAPI was used for nuclear and kinetoplast staining (blue).
