# Supplementary material for: Improved serodiagnosis of Trypanosoma vivax infections in cattle reveals high infection rates in the livestock regions of Argentina
Source: PLoS Negl Trop Dis. 2024 Jun 26;18(6):e0012020. doi: 10.1371/journal.pntd.0012020 (PMC11233006; doi:10.1371/journal.pntd.0012020)
Supplement: S7 Fig — a) Cross-reactivity with the fusion protein MBP. b) Cross-reactivity with samples from cattle infected with Babesia bovis (20), Anaplasma marginale (20), and Trypanosoma theileri (8). Cutoff values of the tests were 0.80 (indicated by broken lines). (PDF) [file pntd.0012020.s007.pdf]

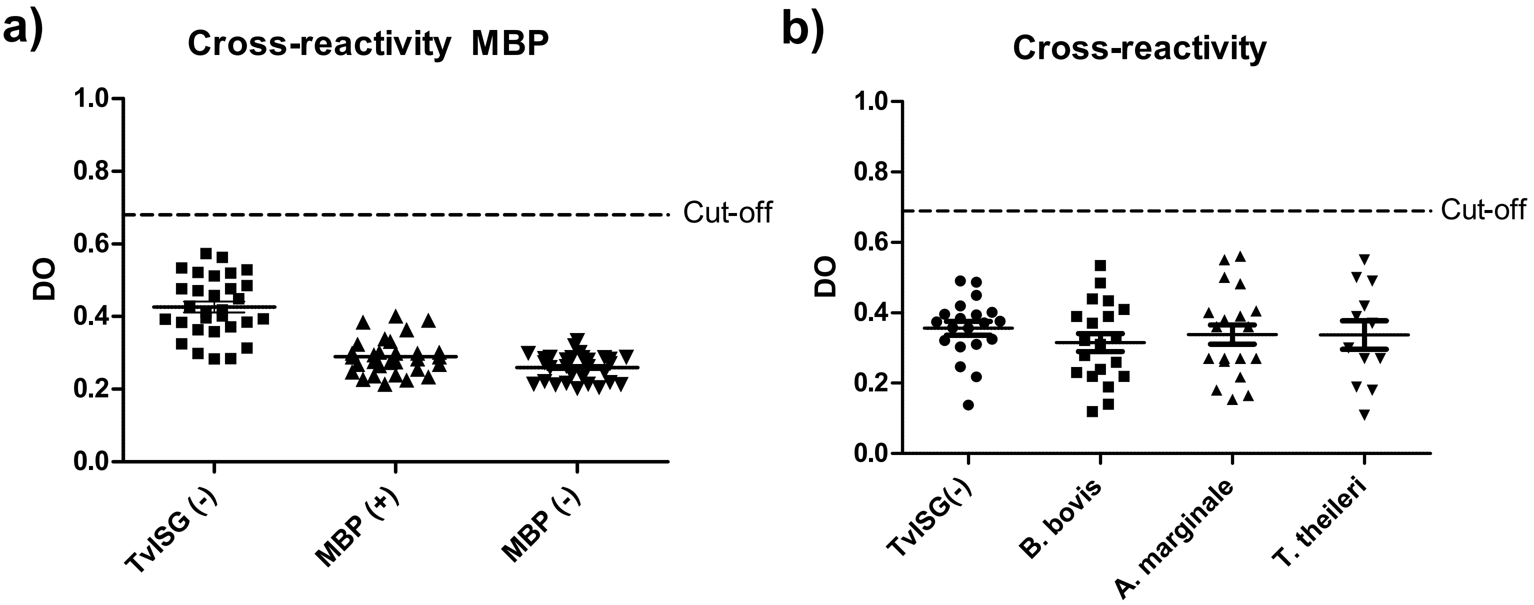

S7 Fig. Evaluation of cross-reactivity in TvISG ELISA. a) Cross-reactivity with the fusion protein MBP. **b)** Cross-reactivity with samples from cattle infected with Babesia bovis (20), Anaplasma marginale (20), and Trypanosoma theileri (8). Cutoff values of the tests were 0.80 (indicated by broken lines).
