# Supplementary material for: Improved serodiagnosis of Trypanosoma vivax infections in cattle reveals high infection rates in the livestock regions of Argentina
Source: PLoS Negl Trop Dis. 2024 Jun 26;18(6):e0012020. doi: 10.1371/journal.pntd.0012020 (PMC11233006; doi:10.1371/journal.pntd.0012020)
Supplement: S1 Table — (PDF) [file pntd.0012020.s008.pdf]

PRIMER	SEQUENCE	RESTRICTION SITE
**TvISGAfFow**	GAATTCATTGAAAAGGTTAAAACTGAAC	*Eco*RI
**TvISGAf Rev**	AAGCTTTCATATTGTGGGAACAACCTTTTC	*Hind*III
**TvISGAm Fow**	GGATCCGAATTCATTGAAAAGGTTAAAACTG	*Bam*HI
**TvISGAm Rev**	AAGCTTGCCTGCAGCGTCAGACGCGACGCTC	*Hind*III

S1 Table: Primers used for amplifying the encoding sequences of Invariant Surface Glycoprotein from African and American *T. vivax*.
